# Use of Electronic Health Record Patient Portal Accounts Among Patients With Smartphone-Only Internet Access

**DOI:** 10.1001/jamanetworkopen.2021.18229

**Published:** 2021-07-26

**Authors:** Kea Turner, Oliver Nguyen, Young-Rock Hong, Amir Alishahi Tabriz, Krupal Patel, Heather S. L. Jim

**Affiliations:** 1 Department of Health Outcomes and Behavior, Moffitt Cancer Center, Tampa, Florida; 2 Department of Health Outcomes and Biomedical Informatics, University of Florida, Gainesville; 3 Department of Health Services Research, Management, and Policy, University of Florida College of Public Health and Health Professions, Gainesville; 4 Department of Head and Neck Endocrinology, Moffitt Cancer Center, Tampa, Florida

## Abstract

This cross-sectional study assesses data from the 2017-2020 Health Information National Trends Survey to examine whether smartphone-only internet access is associated with patient portal use.

## Introduction

Unequal access to information and communication technology, or the digital divide, is a key determinant of patient portal adoption.^1,2^ The digital divide stems from many factors, such as portal usability, digital literacy, internet access, and high broadband costs.^1,2^ The latter have led many US residents (approximately 20%) to opt for smartphone-only internet access, especially individuals from minority racial/ethnic groups and adults with low income.^3^ Smartphone-only internet access could bridge the digital divide in patient portals by providing internet access, or it could exacerbate disparities, given challenges with mobile portal access (eg, data usage limits, lack of mobile-friendly sites).^4^ To address this gap, this study examined whether smartphone-only internet access was associated with patient portal use.

## Methods

For this cross-sectional study, data from January 2017 through June 2020 were obtained from the 2017-2020 Health Information National Trends Survey, which includes questions about patient portal use and internet access in the last 12 months. The survey assesses factors associated with patient portal use, including health care access (eg, insurance coverage), digital literacy (eg, use of internet to view health information), demographic details (eg, income), and health characteristics (eg, comorbidities). We selected factors that are independently associated with patient portal usage.^1,2^

We compared smartphone-only internet access and patient portal use based on sample characteristics, using the Pearson χ^2^ test. We ran 2 multivariable logistic regressions controlling for year to examine which factors were associated with smartphone-only internet access and patient portal use. We removed individuals with missing data or no internet access, or who did not report a health care visit in the last 12 months. We checked for multicollinearity across covariates. We used sampling and jackknife replicate weights to account for the stratified survey design and develop nationally representative estimates. Analyses were conducted in Stata, version 16 (StataCorp). A 2-sided *P* = .05 was used to determine statistical significance. This study was exempted by the Advarra institutional review board because of the use of publicly available data. The results are reported in accordance with the Strengthening the Reporting of Observational Studies in Epidemiology (STROBE) reporting guideline for cross-sectional studies.^5^

## Results

The sample contained 8790 adults. Most participants were women (5196 [59.1%]), non-Hispanic White (6325 [72.0%]), and aged 35 to 49 years (1964 [22.3%]) or 50 to 64 years (2995 [34.1%]). The number of US residents with smartphone-only internet access increased from 21.6% in 2017 to 31.1% in 2020 (*P* < .001) (Table 1). Patient portal use increased from 44.0% (766 of 1742 respondents) in 2017 to 54.8% (1208 of 2204 respondents) in 2020 (*P* < .001). Controlling for other factors, non-Hispanic Black participants (odds ratio [OR], 1.32; 95% CI, 1.01-1.72) and Hispanic participants (OR, 1.33; 95% CI, 1.04-1.72) had significantly higher odds of smartphone-only internet access compared with non-Hispanic White participants (Table 2). Individuals in the highest income category (≥$75 000) had significantly lower odds of smartphone-only internet access compared with those with lower income (<$20 000) (OR, 0.57; 95% CI, 0.40-0.80). Controlling for other factors, smartphone-only internet access was associated with significantly lower odds of portal use compared with having a wired connection (OR, 0.82; 95% CI, 0.74-0.91). After controlling for smartphone-only internet access, higher income (≥$75 000) was associated with significantly higher odds of portal use compared with that for individuals with lower income (<$20 000) (OR, 1.93; 95% CI, 1.62-2.30).

**Table 1.** Sample Characteristics, 2017-2020

Characteristic	Overall, No. (%) (N = 8790)	Smartphone-only internet access, No. (%) (n = 2444)	*P* value	Patient portal adoption, No. (%) (n = 4379)	*P* value
Uses internet to view health information					
Yes	7446 (84.7)	2094 (28.1)	.12	4013 (53.9)	<.001
No	1344 (15.3)	350 (26.0)		366 (27.2)	
Usual source of care^a^					
Yes	6915 (78.7)	1807 (26.1)	<.001	3687 (53.3)	<.001
No	1875 (21.3)	637 (34.0)		692 (36.9)	
Uninsured					
Yes	264 (3.0)	95 (36.0)	.003	71 (26.9)	<.001
No	8526 (97.0)	2349 (27.5)		4308 (50.5)	
Age, y					
18-34	1308 (14.9)	625 (47.8)	<.001	647 (49.5)	.001
35-49	1964 (22.3)	580 (29.5)		997 (50.8)	
50-64	2995 (34.1)	770 (25.7)		1507 (50.3)	
65-74	1801 (20.5)	368 (20.4)		921 (51.1)	
≥75	722 (8.2)	101 (14.0)		307 (42.5)	
Sex					
Men	3594 (40.9)	727 (20.2)	<.001	1651 (45.9)	<.001
Women	5196 (59.1)	1717 (33.0)		2728 (52.5)	
Race/ethnicity					
Non-Hispanic White	6325 (72.0)	1613 (25.5)	<.001	3263 (51.6)	<.001
Non-Hispanic Black	1556 (17.7)	514 (33.0)		723 (46.5)	
Hispanic	909 (10.3)	317 (34.9)		393 (43.2)	
Other	839 (9.5)	267 (31.8)		409 (48.7)	
Income, $					
<20 000	965 (11.0)	369 (38.2)	<.001	332 (34.4)	<.001
20 000-34 999	960 (10.9)	289 (30.1)		361 (37.6)	
35 000-49 999	1083 (12.3)	325 (30.0)		502 (46.4)	
50 000-74 999	1703 (19.4)	489 (28.7)		834 (49.0)	
≥75 000	4079 (46.4)	972 (23.8)		2350 (57.6)	
Education					
High school diploma or less	3863 (43.9)	1147 (29.7)	<.001	1607 (41.6)	<.001
College degree	2832 (32.2)	799 (28.2)		1523 (53.8)	
Postgraduate	2095 (23.8)	498 (23.8)		1249 (59.6)	
Married					
Yes	5211 (59.3)	1334 (25.6)	<.001	2759 (52.9)	<.001
No	3579 (40.7)	1110 (31.0)		1620 (45.3)	
Rural residence					
Yes	973 (11.1)	298 (30.6)	.04	410 (42.1)	<.001
No	7817 (88.9)	2146 (27.5)		3969 (50.8)	
Multiple chronic conditions^b^					
Yes	2639 (30.0)	717 (27.2)	.38	1352 (51.2)	.08
No	6151 (70.0)	1727 (28.1)		3027 (49.2)	
Deafness or hearing impairment					
Yes	534 (6.1)	122 (22.8)	.008	247 (46.3)	.09
No	8256 (93.9)	2322 (28.1)		4132 (50.0)	
Year					
2017	1742 (19.8)	377 (21.6)	<.001	766 (44.0)	<.001
2018	1818 (20.7)	476 (26.2)		810 (44.6)	
2019	3026 (34.4)	905 (29.9)		1595 (52.7)	
2020	2204 (25.1)	686 (31.1)		1208 (54.8)	

^a^ Refers to having a particular clinician whom a patient consults when health care is needed (eg, primary care clinician).

^b^ Having 2 or more chronic conditions (diabetes, hypertension, heart condition, chronic lung disease, and depression or anxiety disorder).

**Table 2.** Factors Associated With Smartphone-Only Internet Access and Patient Portal Use, 2017-2020

Variable	Smartphone-only internet access (n = 8790)		Patient portal use (n = 8790)	
	OR (95% CI)	*P* value	OR (95% CI)	*P* value
Smartphone only				
Yes	NA	NA	0.82 (0.74-0.91)	<.001
No	NA	NA	1 [Reference]	NA
Uses internet to view health information				
Yes	0.92 (0.67-1.26)	.60	2.66 (2.33-3.05)	<.001
No	1 [Reference]	NA	1 [Reference]	NA
Usual source of care^a^				
Yes	0.85 (0.70-1.04)	.12	1.90 (1.69-2.13)	<.001
No	1 [Reference]	NA	1 [Reference]	NA
Uninsured				
Yes	0.87 (0.54-1.41)	.58	0.51 (0.38-0.68)	<.001
No	1 [Reference]	NA	1 [Reference]	NA
Age, y				
18-34	1 [Reference]	NA	1 [Reference]	NA
35-49	0.37 (0.30-0.46)	<.001	0.92 (0.79-1.07)	.28
50-64	0.31 (0.25-0.39)	<.001	0.92 (0.80-1.06)	.26
65-74	0.24 (0.19-0.30)	<.001	0.95 (0.81-1.12)	.57
≥75	0.12 (0.09-0.17)	<.001	0.69 (0.56-0.85)	.001
Sex				
Men	0.48 (0.40-0.58)	<.001	0.69 (0.63-0.76)	<.001
Women	1 [Reference]	NA	1 [Reference]	NA
Race/ethnicity				
Non-Hispanic White	1 [Reference]	NA	1 [Reference]	NA
Non-Hispanic Black	1.32 (1.01-1.72)	.04	0.92 (0.81-1.05)	.21
Hispanic	1.33 (1.04-1.72)	.03	0.85 (0.73-0.98)	.03
Other	0.80 (0.60-1.08)	.15	1.00 (0.85-1.17)	.97
Income, $				
<20 000	1 [Reference]	NA	1 [Reference]	NA
20 000-34 999	0.62 (0.43-0.90)	.01	1.06 (0.88-1.29)	.53
35 000-49 999	0.61 (0.43-0.88)	.009	1.50 (1.24-1.82)	<.001
50 000-74 999	0.60 (0.45-0.81)	.001	1.56 (1.31-1.87)	<.001
≥75 000	0.57 (0.40-0.80)	.001	1.93 (1.62-2.30)	<.001
Education				
≤HS diploma	1 [Reference]	NA	1 [Reference]	NA
College degree	0.86 (0.71-1.05)	.14	1.35 (1.21-1.50)	<.001
Postgraduate	0.72 (0.58-0.89)	.002	1.51 (1.34-1.70)	<.001
Married				
Yes	1.00 (0.83-1.20)	.98	1.11 (1.01-1.23)	.04
No	1 [Reference]	NA	1 [Reference]	NA
Rural residence				
Yes	1.13 (0.89-1.45)	.32	0.76 (0.66-0.88)	<.001
No	1 [Reference]	NA	1 [Reference]	NA
Multiple chronic conditions^b^				
Yes	1.25 (1.02-1.52)	.03	1.29 (1.17-1.44)	<.001
No	1 [Reference]	NA	1 [Reference]	NA
Deafness or hearing impairment				
Yes	0.86 (0.57-1.29)	.46	1.01 (0.83-1.22)	.93
No	1 [Reference]	NA	1 [Reference]	NA
Year				
2017	1 [Reference]	NA	1 [Reference]	NA
2018	1.26 (1.01-1.57)	.04	1.07 (0.93-1.23)	.33
2019	1.44 (1.14-1.82)	.003	1.56 (1.37-1.77)	<.001
2020	1.68 (1.29-2.19)	<.001	1.73 (1.51-1.98)	<.001
Constant	1.89 (1.21-2.95)	.006	0.13 (0.10-0.16)	<.001

Abbreviations: HS, high school; NA, not applicable; OR, odds ratio.

^a^ Usual source of care refers to having a particular provider whom a patient consults when health care is needed (eg, primary care clinician).

^b^ Multiple chronic conditions means having 2 or more chronic conditions (diabetes, hypertension, heart condition, chronic lung disease, and depression or anxiety disorder).

## Discussion

As of 2020, 1 in 4 US residents reported having smartphone-only internet access, which was negatively associated with patient portal use. This study was conducted after the passage of the 21st Century Cures Act, which aimed to enhance mobile portal access,^6^ suggesting further work is needed to optimize such access. After accounting for smartphone-only internet access, some patients (eg, those with lower income) were still less likely to use portals, suggesting multimodal strategies are needed for overcoming the digital divide. Recent policy initiatives aimed at expanding broadband access will likely alleviate some digital barriers; however, other strategies (eg, technology training) are still needed.

This study has several limitations, including the inability to account for language preference—a key barrier to portal adoption—and the use of self-reported data. Nonetheless, it offers important implications for how smartphone-only internet access may affect the digital divide in patient portal use.
